# The pupil response to perceptual switches: What happens when you ignore them

**DOI:** 10.1167/jov.25.8.5

**Published:** 2025-07-03

**Authors:** Bobicheng Zhang, Vasilii Marshev, Jan W. Brascamp

**Affiliations:** 1 Department of Psychology, Michigan State University, East Lansing, MI, USA

**Keywords:** bistable perception, structure-from-motion, pupillometry, eye movement, optokinetic nystagmus

## Abstract

The pupil has been found to dilate after switches in bistable perception, prompting the suggestion that norepinephrine-based neuromodulation plays a causal role in those switches. However, the pupil dilates in response to task-relevant events in general, and, in existing work, perceptual switches were typically task-relevant (e.g., they had to be reported). As such, observed switch-related dilations may have reflected nonspecific task relevance rather than switch-specific processes. Here, we measured pupil responses to perceptual switches that were task-irrelevant. Observers viewed a rotating structure-from-motion sphere consisting of equilateral triangles that inverted at semi-random intervals. In separate conditions, observers either reported perceptual switches (rendering them task-relevant) or reported changes in the triangles' orientation (rendering the switches task-irrelevant). We then used observers’ optokinetic nystagmus to infer perceptual switch moments, even when observers did not report them. Control analyses confirm the reliability of this method. We found that task-relevant switches were followed by pupil dilations, but task-irrelevant ones were not. These results suggest that pupil-associated neuromodulation, although closely linked to task-relevant events, may not have any specific tie with perceptual bistability. These results are consistent with results we recently reported for binocular rivalry, indicating commonality across distinct forms of perceptual bistability.

## Introduction

The pupil, as one of the earliest stops along the path from the environment to the brain, responds to variations in illumination level but its size is also modulated by several affective-cognitive factors such as elevated memory load (Kahneman & Beatty, 1966), attention (Wierda, van Rijn, Taatgen, & Martens, 2012; Wang & Munoz, 2015), and emotional arousal (Bradley, Miccoli, Escrig, & Lang, 2008). During bistable perception, when ambiguous sensory input that could be interpreted in two equally valid manners results in stochastic switching between two perceptual interpretations, pupillary signatures could provide insights regarding neural mechanisms underlying this switching phenomenon.

Perceptual switches during bistable perception have been linked to multiple mechanisms, and one view holds that these switches are closely associated with norepinephrine release from the brainstem (Einhäuser, Stout, Koch, & Carter, 2008). This notion was partly motivated by the finding of a transient pupil dilation accompanying perceptual switches. One known cause of transient pupil dilations is increased norepinephrine release from the locus coeruleus (Ashton-Jones & Cohen, 2005; Joshi, Kalwani, & Gold, 2016), thus leading to the proposal that such norepinephrine release plays a role in shaping perception during bistability.

However, in studies that show pupil dilations accompanying perceptual switches, those switches are usually task-relevant (Einhäuser et al., 2008; Hupé, Lamirel, & Lorenceau, 2009). For instance, whenever the observer experiences a perceptual switch, they may be required to press a key to report the event. Brainstem norepinephrine release is typically associated with elevated effort, alertness, or arousal due to, for instance, the occurrence of an external event of interest, and the same is true of transient pupil dilations (Beatty, 1982; Mathôt, 2018; Joshi & Gold, 2020). Likewise, locus coeruleus shows little or no response to stimuli when they are task-irrelevant (Aston-Jones, Rajkowski, Kubiak, & Alexinsky, 1994; Usher, Cohen, Servan-Schreiber, Rajkowski, & Aston-Jones, 1999; Rajkowski, Majczynski, Clayton, & Aston-Jones, 2004). As such, based on existing work, it is often difficult to distinguish whether observed pupil dilations accompanying perceptual switches inform about the underlying mechanisms of switches (for instance, involving norepinephrine release) or whether those dilations reflect downstream consequences of the switches (alerting, task-execution, etc.). Support for the latter option comes from an earlier study where we rendered perceptual switches task-irrelevant. As a result, the primary switch-related pupil response was no longer a dilation but a constriction (Brascamp, De Hollander, Wertheimer, DePew, & Knapen, 2021).

In the present study, we examine the generality of the findings of Brascamp et al. (2021) on pupil constriction associated with task-irrelevant perceptual switches. In the earlier study, the bistable stimulus used was a binocular rivalry stimulus: Brascamp et al. (2021) presented two planes of dots dichoptically (i.e., one to each eye). The two planes moved in opposite directions and had different colors, resulting in perceptual switches between the two planes. Although the constriction finding has not been evaluated outside of that study, the finding of pupil dilations associated with task-relevant switches is much more established: Einhäuser et al. (2008) reported it for a variety of stimuli ranging from ambiguous figures to auditory stimuli, and later it was confirmed for several other paradigms (Hupé et al., 2009; Kloosterman et al., 2015). There is some reason to believe that binocular rivalry may yield different results than other perceptual bistability paradigms. Binocular rivalry is often thought to be resolved at relatively early visual processing stages (Blake & Logothetis, 2002; Wunderlich, Schneider, & Kastner, 2005; Tong, Meng, & Blake, 2006) and is considered more stimulus-driven than other forms of perceptual bistability (Meng & Tong, 2004). Indeed, perception of several of the stimuli used by Einhäuser et al. (2008) is susceptible to higher-level influences such as attention (Meng & Tong, 2004; Scocchia, Valsecchi, & Triesch, 2014) and voluntary control (Toppino, 2003; Brouwer & van Ee, 2006), suggesting a substrate that includes higher parts of the cortical processing hierarchy. Of note, transient pupil dilations can signal alterations in attention (Nieuwenhuis, Geus, & Jones, 2011; Wang & Munoz, 2015) or voluntary effort (Kahneman, Tursky, Shapiro, & Crider, 1969; van der Wel & van Steenbergen, 2018). Based on considerations such as these, we deemed it possible that the Brascamp et al. (2021) finding of switch-related pupil constriction, in contrast to the more general finding of switch-related pupil dilation, might not be entirely attributable to the difference in task relevance, but could also in part reflect stimulus differences. In the present study, therefore, we examine how the pupil responds to task-irrelevant perceptual switches when they arise in a bistable perception paradigm other than binocular rivalry. In particular, we used a structure-from-motion stimulus similar to one reported to elicit pupil dilations in the case of task-relevant switches (Einhäuser et al., 2008).

A practical issue when interested in concomitants of task-irrelevant perceptual switches is that the moments of switching cannot be inferred by asking observers to report them (as this would render the switches task-relevant). To detect the occurrence of switches without asking observers to report them, we relied on optokinetic nystagmus (OKN), a type of spontaneous eye movement exhibited while viewing moving objects (Leopold, Maier, & Logothetis, 2003). OKN has been used for this purpose in other work, the basic idea being that if competing percepts of a bistable stimulus are associated with different directions of perceived motion, the direction of gaze displacement can signal which percept is being experienced (Fox, Todd, & Bettinger,1975; Naber, Frässle, & Einhäuser, 2011; Frässle, Sommer, Jansen, Naber, & Einhäuser, 2014; Wilbertz, Ketkar, Guggenmos, & Sterzer, 2018; Brascamp et al., 2021). Thus in the present study the use of OKN enabled us to include conditions during which perceptual switches were task-irrelevant yet still identify the switches’ occurrence. This, in turn, allowed us to compare pupil signatures of task-irrelevant switches to those of task-relevant switches.

## Methods

### Participants

This study was approved by the Institutional Review Board at Michigan State University. Fifty-nine subjects participated. All were undergraduate students who registered for the study on the HPR/SONA system in exchange for psychology course credit. Informed consent was obtained from all participants before the study, and a debriefing letter clarifying its purposes was provided at the end.

### Stimulus

**Figure 1.**
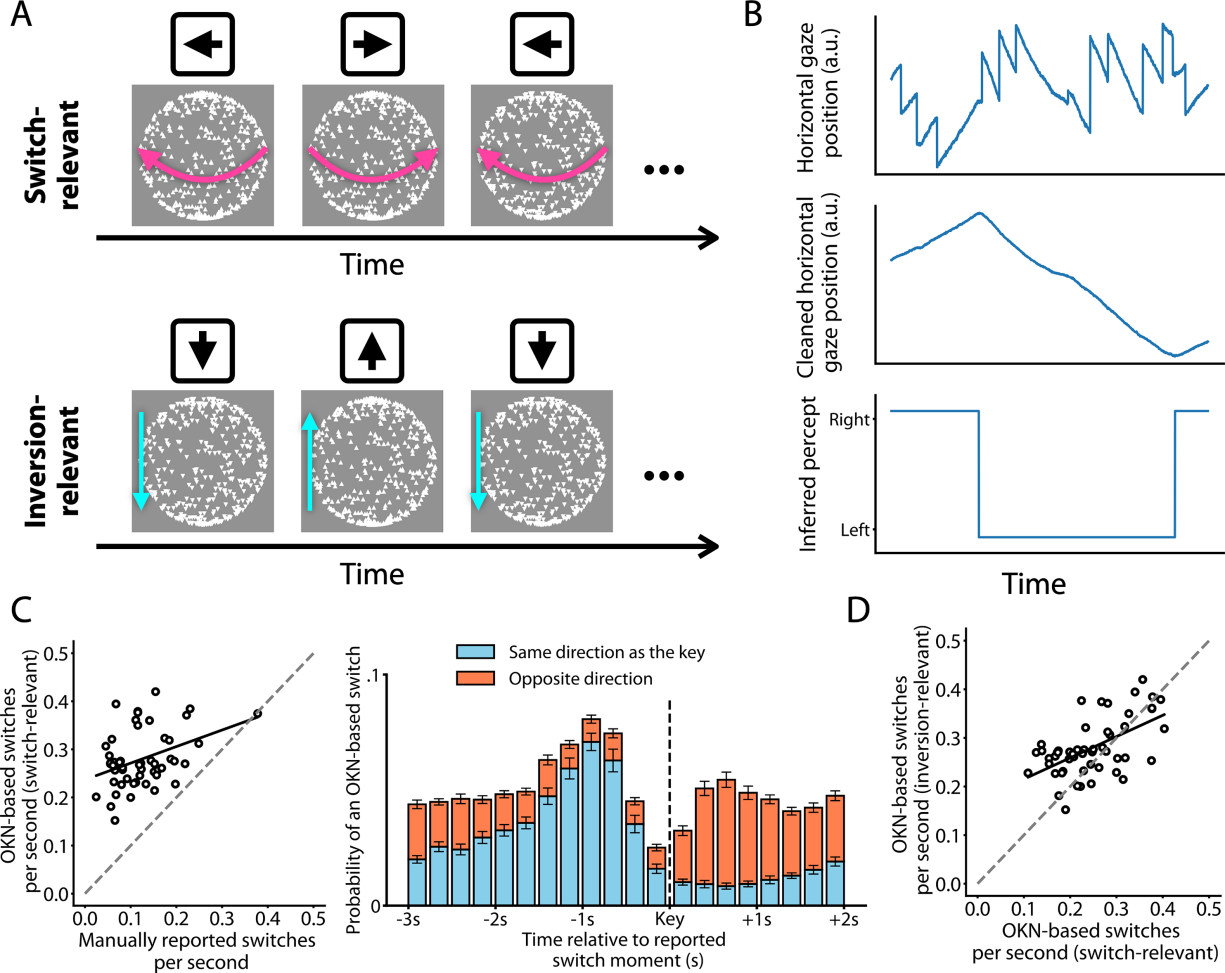
Experimental design and OKN validation. (**A**) The two tasks that observers were instructed to complete included pressing left or right arrow keys to indicate rotation direction (top) and pressing up or down arrow keys to indicate the orientation of triangles. Note that the stimulus was the same in both tasks, meaning the only difference was the task. (**B**) Moments at which perceptual switches occur were identified based on OKN during experimental trials. (**C**, left) In the switch-relevant condition, the rate of perceptual switches based on OKN correlates positively with that based on manual reports. (**C**, right) There was also a temporal correspondence, such that OKN-based switches tended to occur 0.75 to 1.5 seconds before manually reported switches toward the same rotation direction (blue bars). Each bar represents a 250-ms window relative to the moment of the key press (dashed line). The error bars represent the standard error of the mean. (**D**) The rate of perceptual switches based on OKN is correlated between switch-relevant and inversion-relevant conditions.

The current study used a structure-from-motion rotating sphere that could be perceived as rotating in one direction at times and in the opposite direction at other times (Figure 1A). The sphere was presented on a cathode ray tube monitor, with the screen resolution set to 1024 × 768 pixels and the refresh rate to 75 Hz. The sphere had an on-screen diameter of 6° of visual angle at a 60-cm viewing distance and consisted of 400 randomly placed slow-moving white equilateral triangles. Each triangle had a lifetime of 500 ms (and a random starting “age”), after which it was replaced by a triangle elsewhere. The rotation speed was maintained at one complete cycle every six seconds. We achieved the illusion of a transparent rotating sphere by placing each triangle randomly on the surface of the simulated rotating sphere, which means that each triangle had a sinusoidal speed profile (fastest speed when a triangle crosses the sphere's vertical midline and a direction reversal at each sphere edge). This results in the impression of a transparent sphere of which both the front surface moving in one direction and the back surface moving in the opposite direction are simultaneously visible. However, it is ambiguous which surface of the sphere is the front surface and which is the back surface. In many existing studies, the result is that the perceived direction of rotation fluctuates over time (e.g., Nawrot & Blake, 1989). In one of our two conditions, observers reported switches in this perceived direction, thus rendering the switches task-relevant. In the second condition, the switches were task-irrelevant, and observers had an unrelated task. This task was designed to keep observers’ attention on the ambiguous sphere but not on its direction of rotation. In particular, in this second condition, observers reported changes in the orientation of the triangles that formed the sphere. In both conditions, the orientation of all triangles was randomly set to point either upward or downward at every onset of the sphere and then inverted at random moments while the sphere was on the screen (on average once every five seconds; see Supplementary Figure S1); events that observers reported in the second condition.

### Procedure

The experiment was programmed using PsychoPy (version 2021.2.3) (Peirce et al., 2019). Subjects performed the tasks in our laboratory. To control viewing distance at 60 cm, they were asked to keep their heads on a chin rest with their foreheads pressed against a forehead bar during the experiment. The height of the chin rest was adjusted depending on individual anatomy, keeping the position of the eyes maximally constant across observers. The eye tracker, an SR Research EyeLink 1000 Plus, was positioned directly in front of the cathode ray tube monitor but at a lower height to not block any portion of the screen. The sampling rate was set to 1000 Hz.

The current study used a within-subjects design and two separate conditions. In the so-called “switch-relevant” condition, observers were instructed to pay attention to the sphere's rotation direction and report in real-time any reversals in the perceived direction of rotation by pressing either the left arrow key or the right arrow key (Figure 1A, top). In the so-called “inversion-relevant” condition, on the other hand, observers were instructed to pay attention to the orientation of the triangles and report in real-time any reversals in the orientation of the triangles using either the up arrow key or the down arrow key (Figure 1A, bottom). The order of the two conditions was randomized between observers, and observers were not informed about the nature of their second condition until they had completed the first. Before each condition, observers completed one practice trial. In the practice trial of the second condition, to help observers readjust to the new task, feedback was provided if they accidentally pressed a key associated with the previous condition. In each condition, observers were also told that any changes other than those being reported would be irrelevant.

### Data analysis

Four observers were excluded for failure to follow the instructions in one of the two conditions. Specifically, three observers were excluded based on the switch-relevant condition: less than half of their total key presses in that condition indicated a different rotation direction from the previous key (i.e., a perceptual switch). For these observers, respectively, only 34%, 44%, and 46% of their key presses met this criterion, while this percentage averaged 82% (*Min* = 56%, *Max* = 96%, *Mdn* = 85%) for the remaining observers. One observer was excluded based on the inversion-relevant condition, namely for spamming keys in that condition, resulting in five times more key presses than triangle inversions.

#### Extracting perceptual switches

In the switch-relevant condition, during which observers pressed the left or the right arrow keys to indicate the perceived motion direction, all key presses that indicated the opposite direction from the previous key press were interpreted as indicating perceptual switches. All key presses that repeated the direction of the previous key press were ignored.

In both the inversion-relevant and switch-relevant conditions, perceptual switch moments were inferred based on the direction of gaze displacement during OKN. We used a similar approach as previous studies (Naber et al., 2011; Frässle et al., 2014; Aleshin, Ziman, Kovács, & Braun, 2019; Brascamp et al., 2021). The basic idea is as follows. OKN is characterized by ongoing alternations between a slow phase during which the on-screen gaze position gradually moves in the same direction as a moving visual stimulus (in the present case, its perceived direction) and a fast phase during which a saccade rapidly displaces the gaze position in the opposite direction (Leopold et al., 2003). By algorithmically removing the fast phases and then examining the direction of the remaining gradual gaze displacement, one can identify moments when this direction reverses and tentatively mark those moments as corresponding to reversals in the perceived direction (Figure 1B).

Data preprocessing followed very similar steps as in Brascamp et al. (2021), which means that saccades were identified for each eye independently using the saccade identification algorithm by Engbert and Mergenthaler (2006) and then combined across eyes, and that we also identified periods of blinks and missing data to set gaze displacement during those periods to zero. Gaze displacement during saccades was also set to zero, thus resulting in a trace documenting only displacements during the OKN slow phases (Figure 1B, top to center panel). The only substantial difference with the Brascamp et al. (2021) methods is that we did not use the Eyelink preprocessing software to identify blinks but rather identified blinks as periods that started with an unusually rapid reduction in pupil size (an artifact of the upper eyelid moving over the pupil) and ended with an unusually rapid increase (the eyelid moving back up).

After preprocessing the gaze position signal in this fashion, we quantified the direction of gaze displacement for a 1500-ms window that was moved over the time series in steps of 38 ms. In each time window, we first fit a simple linear curve to the vertical gaze position as a function of time and another to the horizontal gaze position as a function of time. The arctangent of the two slopes quantified the gaze displacement angle within that time window: the direction in which the center of gaze moved across the screen. To avoid long periods with missing or artifactual signals contaminating our calculation of gaze displacement angle near blinks, all time points that fell between 250 ms before the start of a blink and 400 ms after the end of a blink were assigned an average gaze displacement value from nearby time points outside of this window. This average value was computed across the 100 ms immediately before the 250-ms pre-blink buffer and the 100 ms immediately after the 400-ms post-blink buffer. We then assigned a perceived motion direction to each time window based on the cosine value of the gaze displacement angle within the corresponding time window: any window with a cosine smaller than −0.7 was tentatively assigned a leftward perceived motion of the front surface, and the same for 0.7 and rightward perceived motion. Windows with intermediate cosine values were left unassigned. Finally, each time point midway between two opposite assigned percepts was marked as a perceptual switch.

#### Pupil size signal

**Figure 2.**
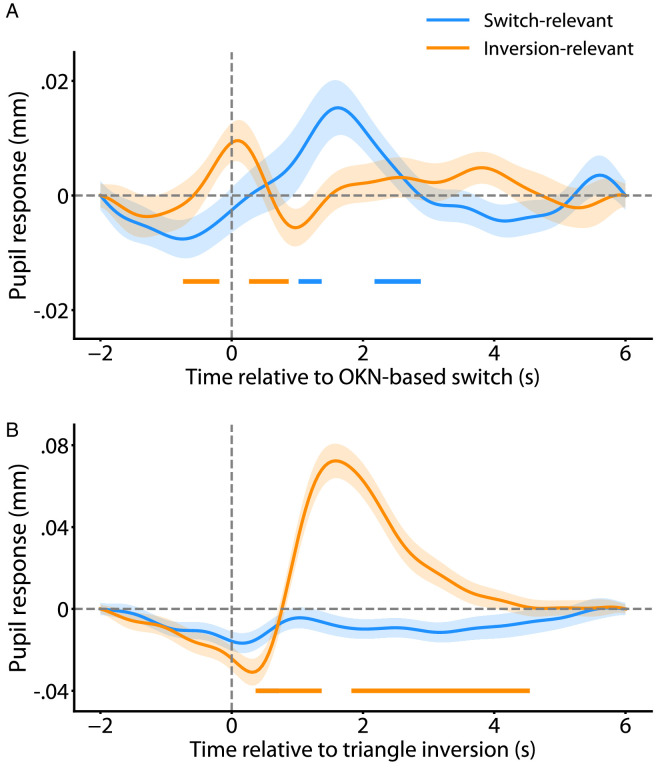
Pupil responses time locked to OKN-based perceptual switch (**A**) and triangle inversion (**B**). (**A**) Change in pupil diameter as a function of time around the moment of the perceptual switch for both conditions. (**B**) Change in pupil diameter around the moment of the triangle inversion for both conditions. The shaded regions represent standard error of the mean. Colored bars at the bottom correspond to time points during which the rate of change in pupil size is significantly different compared to baseline (cluster-level *p* < 0.01).

Pupil size analysis followed the same methods as Brascamp et al. (2021). This means that pupil size was linearly interpolated across blinks and missing data intervals and that slow drifts in pupil size were removed. Specifically, for each trial, we applied a 15-s sliding boxcar window to the pupil size signal, at each time point computing the average within the window, thereby producing a smooth version of the time series. Gradual changes in the pupil size, but not faster, fluctuations, were then removed by subtracting this smooth version from the original signal. It also means that to quantify pupil responses associated with specific events, such as perceptual switches, we used a general linear model deconvolution approach in which event-related responses were modeled as a weighted sum of functions from a Fourier series by using the ResponseFitter class from the nideconv package (de Hollander & Knapen, 2018). In both conditions, our design matrix had three regressors: triangle inversions, OKN-based switches, and blinks. For each event in Figure 2, the deconvolution time window ran from two seconds before the event to six seconds after.

#### Statistics

Although we will visualize event-related pupil signals in terms of absolute pupil size, we performed inferential statistics on the temporal derivative of pupil size: on pupil size change rate (cf., Brascamp et al., 2021). Two reasons for this are that neural events are arguably more directly related to changes in pupil size than with particular pupil size values, and that the general linear model approach sometimes leaves it unclear what an appropriate baseline is against which to compare pupil size values (e.g., an event-related pupil size curve may show no deflection at all, yet hang significantly below 0 mm throughout its entire duration). As such, we computed across-observer pupil size change rate curves by taking the temporal derivative of individual-observer event-related pupil size curves, and then averaged the result. To test where this average derivative curve deviated from 0 mm/s (i.e., to test at which time points pupil size significantly changed) while avoiding a multiple comparison problem, we followed an approach identical to Brascamp et al. (2021). This approach involves computing cluster-level significance instead of per-time point significance (Bullmore et al., 1999). First, we performed single-sample *t*-tests for each time point across observers and used the results to identify clusters of consecutive time points with *p* < 0.01 that all shared the same sign of pupil size change. For each of the observed clusters, we then calculated its mass, or the sum of t-scores from all time points within that cluster. Then, we performed a permutation analysis to identify the statistical significance of such cluster mass values. In particular, on each of 1000 iterations, the sign of each observer's peri-event pupil change data was randomly inverted or not (50% chance), and across-observer cluster masses were computed as described above for the real data. For each such iteration, the mass of the most extreme cluster was marked down, resulting in 1000 simulated cluster values. Finally, we assigned to each of the observed clusters a Monte Carlo *p* value equal to the proportion of the simulated cluster mass values that were more extreme than the observed cluster mass. All observed clusters with a *p* value smaller than 0.01 were considered significant.

## Results

### OKN validation

Although OKN has been used before for identifying switches in bistable perception, we know of no work doing this for a bistable structure-from-motion stimulus, and it is important to verify that the moments identified by our algorithm are, indeed, switch moments. We computed several metrics to investigate this. First, we found a positive correlation across observers between the rate of manually reported switches and the rate of OKN-based switches in the switch-relevant condition (Pearson's *r* = 0.38, Fig. 1C left). This means that people who are classified as “fast switchers” or “slow switchers” based on their key press reports also tend to be classified as such based on the OKN data, consistent with the notion that the OKN method, indeed, tends to mark perceptual switch moments. Do note that the correlation is not perfect, so it is quite possible that the OKN method also marks some moments that do not feature in the key press record or overlooks moments that do (see Discussion).

Nevertheless, the correlation indicates substantial correspondence between the two methods of inferring switches. As a more specific metric, we also evaluated the temporal alignment between switch moments marked by key presses and those marked by the OKN algorithm in the switch-relevant condition. In particular, for different time points within a time window anchored to key press reports, we tallied up the number of identified OKN-based switches. This analysis revealed a distinct peak in OKN-based switches at approximately 0.75 to 1.5 seconds before manually reported switches (Figure 1C right; the peak in the overall height of the stacked bars). This is consistent with a good temporal alignment between manually reported and OKN-based switches, and a slight motor delay in the former would partly explain the temporal shift (see Brascamp et al., 2021 for similar results)^1^. To provide more detail about the relation between key presses and OKN behavior, the stacked bars of Figure 1C (right) distinguish two different kinds of OKN-based switches. Specifically, if we assume that the eyes follow the sphere surface that is perceived in front, then we can mark OKN-based switches as either matching the accompanying key press report in terms of switch direction (blue) or as not matching that manual report (orange). This reveals that the observed peak in OKN-based switches ahead of key press reports is completely due to an increase in matching switches, while the rate of non-matching switches actually goes down at that time. This again indicates that the OKN method is suitable for identifying perceptual switches and, moreover, supports the idea that gaze tends to follow the direction of the perceived front surface.

Although Figure 1C right critically confirms temporal alignment between OKN-based switches and manually reported switches, it is also important that there is *no* temporal alignment between OKN-based switches and triangle inversions in our inversion-relevant condition. We therefore performed the same analysis using triangle inversions of the inversion-relevant condition as the triggering event along the x-axis (Supplementary Figure S2). We found that, contrary to manually reported perceptual switches, triangle inversions are not systematically associated with OKN-based switches.

The results of Figure 1C converge on the notion that, within the switch-relevant condition, key presses and OKN analysis highlight similar switch moments. We performed one further validation analysis to address the possibility that OKN patterns may depend strongly on an observer's task, such that results obtained in the switch-relevant condition may not generalize to the inversion-relevant condition. In particular, we assessed the across-observer correlation between the rate of OKN-based switches in the switch-relevant condition and the same rate in the inversion-relevant condition. We found a positive correlation (Pearson's *r* = 0.57, Figure 1D), inconsistent with the idea that the OKN-based method performs dramatically better or worse in the inversion-relevant condition, and instead suggesting that the method is similarly effective irrespective of which task the observer was performing. Taken together, these results show that OKN can be used as an indicator of perceptual switches for the structure-from-motion rotating sphere.

### Pupil responses and task relevance

Figure 2A shows pupil diameter as a function of time surrounding OKN-identified perceptual switches (time 0), averaged across observers. Colored bars below the curves indicate periods where pupil size significantly changes over time (i.e., where the change rate deviates significantly from 0 mm/s). We observe a gradual increase in pupil size following perceptual switches in the switch-relevant condition, peaking at around 1.5 to 2 seconds after the identified switch before returning to baseline (Figure 2A, blue curve). This dilation following reported perceptual switches resembles the one observed in similar paradigms in other studies when observers are instructed to report perceptual switches (e.g., Einhäuser et al., 2008). In contrast, in the inversion-relevant condition, the pupil constricts in the period of this dilation and also shows a smaller dilation slightly earlier in time (Figure 2A, orange curve). The constriction resembles the one observed in the Brascamp et al. (2021) study using binocular rivalry, in a condition where switches were similarly task-irrelevant. That study also showed a non-significant trend toward the earlier dilation (see next section). In sum, these results support the idea that when perceptual switches are task-relevant, the pupil dilates following an OKN-based switch, yet when perceptual switches are task-irrelevant, the pupil constricts following an OKN-based switch instead.

For comparison, Figure 2B shows pupil diameter as a function of time surrounding moments at which triangle inversions occur (time 0), averaged across observers. We observe a sharp increase in pupil size following triangle inversions in the inversion-relevant condition (in which perceptual switches are ignored, but triangle inversions are not), peaking at around 1.5 to 2 seconds after the event before returning to baseline (Figure 2B, orange curve). Like the perceptual switch result for the switch-relevant condition (Figure 2A, blue curve), this shows another dilation response following a task-relevant stimulus, in this case, a triangle inversion. On the other hand, the blue curve shows that triangle inversions do not produce a significant pupil response when ignored (in this condition, the observers are, instead, reporting perceptual switches).

### Peri-switch dilation

The above results reinforce the notion (Brascamp et al., 2021) that the pronounced post-switch dilation that has been reported for task-relevant switches for many stimuli is replaced by a constriction when switches are task-irrelevant. However, we were intrigued by the earlier peri-switch dilation right around the time of task-irrelevant switches (Figure 2A, orange curve). We observed a similar dilation (although insignificant) in our earlier work on binocular rivalry. One possible contributing factor here is the influence of task order. As mentioned, the order in which participants completed the two conditions was randomized, meaning that approximately half completed the inversion-relevant condition first. In contrast, the rest completed the inversion-relevant condition second. Those who performed the inversion-relevant condition first had no reason to pay attention to perceptual switches during that condition. However, the others started the inversion-relevant condition after spending substantial time explicitly focusing on perceptual switches and conceivably needing time to readjust to the new task. This would mean that the earlier dilation around the time of the (nominally) ignored perceptual switches in Figure 2A could partly result from observers’ attention inadvertently being drawn to those switches and perhaps even from observers’ efforts to overcome this tendency.

**Figure 3.**
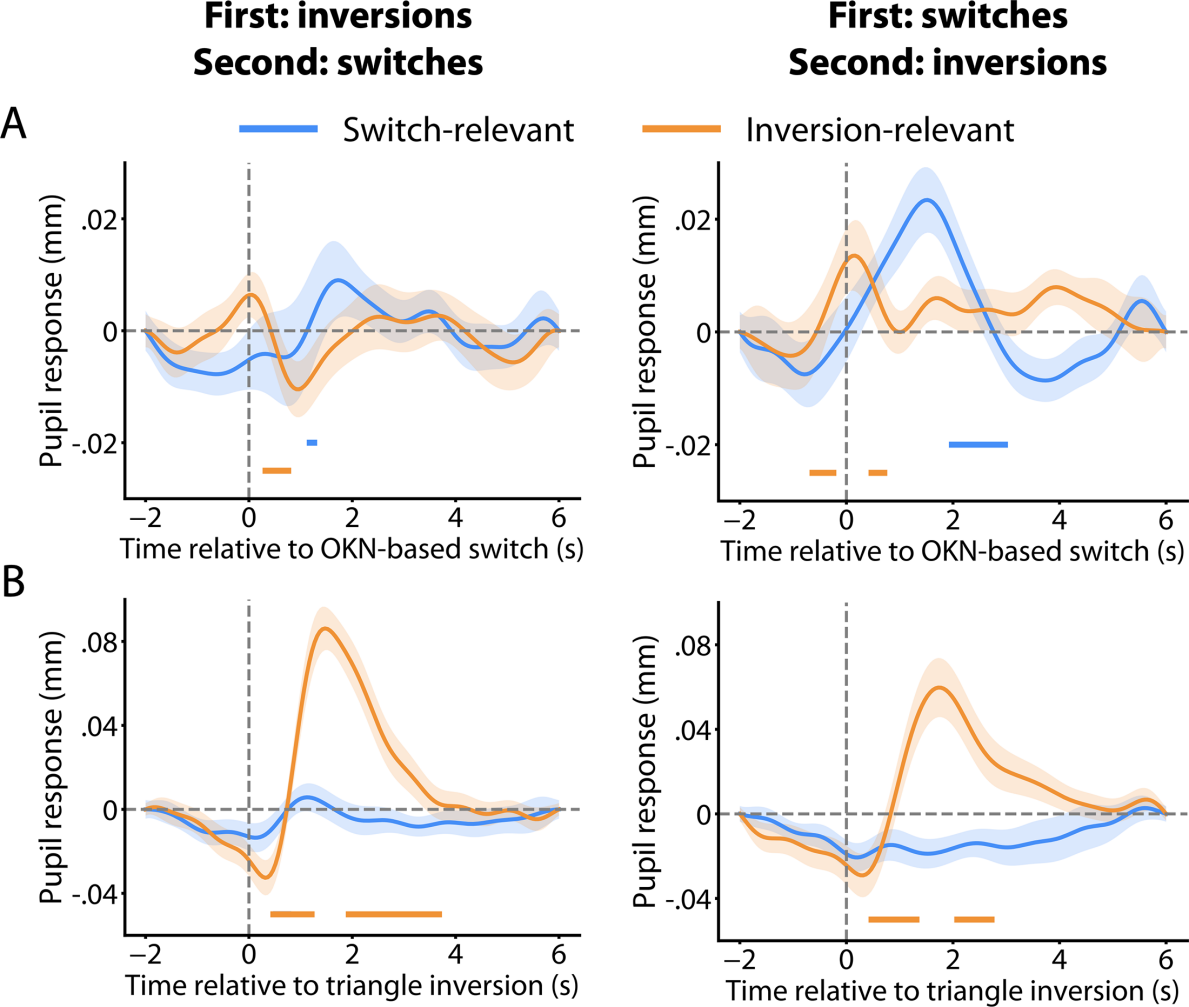
Pupil responses time-locked to OKN-based perceptual switch (**A**) and triangle inversion (**B**) and organized by the order in which observers complete the tasks. A total of 28 observers completed the task of reporting triangle inversions first and reporting perceptual switches second, while the rest followed the opposite order. (**A**) The early dilation around the time of OKN-based switches in the inversion-relevant condition is only present when this condition is performed second. (**B**) There is also a hint of a dilation after triangle inversions in the switch-relevant condition when this condition is performed second, but this pattern does not reach statistical significance. The shaded regions represent the standard error of the mean. Colored bars at the bottom correspond to time points during which the rate of change in pupil size is significantly different compared to baseline (cluster-level *p* < 0.01).

To test this hypothesis, we divided our 52 participants into two groups depending on the order in which they completed the tasks. For completeness, we analyzed the resulting curves, not just for perceptual switches but also for triangle inversions. The results seem generally consistent with the scenario sketched out above: the peri-switch dilation is present for (nominally) ignored switches in participants who just spent time focusing their attention on those switches (Figure 3A, right orange curve), but it is absent (or, at least, not significant) in participants who did not (Figure 3A, left orange curve). In fact, even ignored triangle inversions hint toward dilation in observers who just spent time focusing on those inversions (Figure 3B, left blue curve). Completeness does require us to point out that these are not the only differences between the results for these two groups of participants. Notably, the dilation accompanying task-relevant perceptual switches is more robust if the task of reporting switches is performed first (Figure 3A, right blue curve) than when it is performed second (Figure 3A, left blue curve). Still, these results are consistent with the idea that peri-switch dilation in the inversion-relevant condition is partly due to difficulty ignoring events that needed to be reported in the previous block.

## Discussion

In the present study, we presented a structure-from-motion rotating sphere to observers. We asked them to report either the sphere's rotation direction or the composing triangles' orientation in separate conditions. Our results demonstrate a pupil dilation following actively reported perceptual switches, consistent with prior work (Einhäuser et al., 2008; Hupé et al., 2009; Kloosterman et al., 2015), as well as following actively reported on-screen triangle inversions. However, when perceptual switches become task-irrelevant, the pupil does not dilate following switches but constricts. As such, the present findings extend our earlier result for binocular rivalry (Brascamp et al., 2021) to an ambiguous structure-from-motion stimulus, arguably more susceptible to higher-level cognitive influences. We interpret these findings as evidence that at least the bulk of switch-related dilation found in previous work that involved participants actively reporting switches was due to the switches’ task relevance rather than the nature of switches per se. This interpretation is further supported by the same pattern observed for on-screen triangle inversions: pupil dilation responses were present when inversions were actively reported but not when they were task-irrelevant (Figure 2B). This interpretation echoes earlier conclusions that highlight the influence of task relevance and task execution on switch-related pupil responses (Hupé et al., 2009; Brascamp et al., 2021).

Mechanisms driving perceptual switches during bistability have been a popular subject of inquiry for at least decades. The previously identified transient pupil dilation after (task-relevant) perceptual switches may suggest another potential candidate (Einhäuser et al., 2008; Hupé et al., 2009). Pupil dilation is known to coincide with norepinephrine release from the locus coeruleus (Yoshitomi, Ito, & Inomata, 1985; Ashton-Jones & Cohen, 2005; Joshi et al., 2016); thus the observed dilation response has contributed to the suggestion of a possible link between perceptual switches and dilation-associated norepinephrine modulation of cortical function (Einhäuser et al., 2008; Laeng, Sirois, & Gredebäck, 2012).^2^ However, based on our results, it is likely that at least the bulk of the dilation observed after reported perceptual switches in earlier work was linked to the task relevance of the switch events, as opposed to the mechanisms that modulate perception. Other work is also generally consistent with this idea. One study concluded that the act of reporting contributes to a large portion of the switch-related dilation response (Hupé et al., 2009). Similarly, in work using ambiguous apparent motion stimuli, the pupil dilates immediately after the button press in both bistable and replay conditions (Nakano, Ichiki, Fujikado, 2021). In sum, the finding that task relevance plays such a prominent role in the presence and magnitude of the switch-related dilation response suggests that this dilation may not be primarily informative about the mechanisms that drive perceptual switching but rather that at least the bulk of the dilation is another instance of the well-studied pupil dilations that accompany increased effort and arousal (Beatty, 1982; de Gee et al., 2017; Joshi & Gold, 2020).

The above conclusion notwithstanding, our results show that some reduced dilation remains, even when perceptual switches are task-irrelevant (Figure 2A orange curve). This remaining dilation occurs earlier than the larger dilation that follows task-relevant switches, almost at the exact moment as the switch event. In our previous work, a similar data pattern was present for binocular rivalry (Brascamp, Ee, Noest, Jacobs, & van den Berg, 2021). This earlier residual dilation may relate to the switching mechanism—a possibility worth examining in future research. It is also possible that this dilation, like the larger dilation that follows task-relevant switches, is a downstream consequence of the switch event, reflecting a form of alerting or re-orienting because of the perceptual change. The analyses of Figure 3A are generally consistent with this latter idea by showing that the residual dilation is only significant in our data for participants who have previously learned that these perceptual changes can be important. Of note, the temporal order of these residual dilations relative to the OKN-identified switch events does not conclusively indicate a causal sequence. We find these residual dilations to start before these events by a few hundred milliseconds, and this may suggest that the dilations reflect the switches’ cause. This is a possibility, but it should be noted that perceptual switches are often not instantaneous and commonly unfold over hundreds of milliseconds (Brascamp et al., 2006; Knapen, Brascamp, Pearson, Ee, & Blake, 2011; Naber et al., 2011; Weilnhammer, Ludwig, Hesselmann, & Sterzer, 2013). It is unclear at which moment within such a period the OKN algorithm would mark a switch, so it is uncertain whether a dilation that precedes an OKN-marked switch also precedes the moment at which the actual perceptual change starts. As such, it is unclear what the fine-scale temporal alignment of dilation and OKN-marked switch means with regard to causal order.

When it comes to the pupil response that follows switches, our results indicate that the robust dilation following task-relevant switches turns into a pupil constriction when switches can be ignored. What is the interpretation of this constriction? We have previously speculated (Brascamp et al., 2021) that a similar constriction, observed during binocular rivalry, may result from the changes in visual cortical activity that accompany perceptual switches. Outside of the context of perceptual bistability, isoluminant changes in visual input commonly cause pupil constrictions, for instance, changes in color, spatial frequency, or motion that keep overall illuminance unaltered. These constrictions have been proposed to be due to altered visual cortical activity prompted by the input change (Barbur, Harlow, & Sahraie, 1992; Sahraie & Barbur, 1997). That proposal stipulates that these visual cortical transients temporarily reduce inhibition upon the Edinger-Westphal nucleus (for instance, by the locus coeruleus; Mathöt, 2018; Joshi & Gold, 2020), thereby resulting in decreased pupil size. With regard to perceptual bistability, it has been well-known that perceptual switches during viewing of structure-from-motion, just like switches during binocular rivalry (Leopold & Logothetis, 1996; Tong, Meng, & Blake, 2006), are associated with altered responses in various regions of the visual cortex (Anderson & Bradley, 1998; Dodd, Krug, Cumming, & Parker, 2001; Brouwer & van Ee, 2007). Consequently, we speculate that switch-related constrictions, observed here and in Brascamp et al. (2021), rely on the same route from the cortex to the pupil as has been suggested in relation to constrictions caused by isoluminant input changes (Barbur et al., 1992). If this is true, then these switch-related constrictions may be a useful index of visual cortical responses during perceptual bistability.

We relied on optokinetic nystagmus for inferring perception and for identifying the moments when it switches. To our knowledge, no existing study has used this approach in the context of structure-from-motion stimuli. Instead, OKN has been used for this purpose primarily in the context of binocular rivalry (Fox et al., 1975; Naber et al., 2011; Frässle et al., 2014; Brascamp et al., 2021), as well as for moving plaids (Wilbertz et al., 2018) and transparent motion (Watanabe, 1999). Our results indicate that OKN can be used in this way to study bistable structure-from-motion, as well, but the approach certainly has shortcomings. In particular, for most of our observers, the OKN method identifies a larger number of switches than the observer reports (i.e., the majority of our observers are positioned above the identity line in Figure 1C, left), suggesting that the method spuriously marks some non-switch moments as switches. Aside from spuriously marked switches, an additional reason for the discrepancy could be that some percepts were too brief for manual reports, yet did cause OKN direction reversals (cf., Naber et al., 2011). This is also likely part of the explanation for why the magnitude of the computed pupil dilation response is so much smaller for task-relevant perceptual switches (Figure 2A blue curve) than for task-relevant triangle inversions (Figure 2B orange curve): for the former, the contribution of actual switch moments to the response estimate has been diluted by that of spuriously marked ones. Although our OKN-based algorithm is overall similar to gaze-based methods used for identifying perceptual switches elsewhere, our approach is relatively basic compared to some other approaches, and it is quite possible that the correspondence with manually reported switches can be improved by incorporating components of those other approaches. This includes, rather than using just the gaze displacement angle of the OKN slow phase, combining both slow-phase and fast-phase metrics in a perception classification algorithm (Wilbertz et al., 2018), or using polynomial splines to interpolate missing data in the gaze displacement velocity trace (Aleshin et al., 2019) rather than our basic linear interpolation method.

One factor that may contribute to the spurious marking of switches by our OKN algorithm is that both the front and back surfaces of the “sphere” are visible simultaneously so that, in principle, the eyes may track either surface and may even alternate between surfaces over time. Note that our algorithm would be equally effective for observers who consistently tracked the front surface and those who consistently tracked the back surface (perceptual switches would be marked correctly in both cases), but that alternate tracking of the front and back surfaces would result in spuriously marked switches. Existing work suggests that most observers attend to and ocularly track the front surface in such situations (Watanabe, 1999), and our analyses indicate that this was also true in our case (Figure 1C). Further, by using limited-lifetime sphere elements, we discouraged one specific way of alternating between surfaces: by means of tracking a specific element around the sphere. Still, it is possible that some alternations between tracking the front and back surfaces did happen, and that this accounts for some of the spuriously marked OKN-based switches.

## Conclusions

The pupil is modulated by numerous cognitive and perceptual processes and, therefore, can help characterize mechanisms behind these processes, including perceptual switching as a result of ambiguous visual input. By including a condition during which observers are not attending to perceptual switches, the current study allows most of the behavioral consequences of perceptual switches to be separated from the switch events themselves. Our results demonstrate that the pupillary signature after perceptual switches consists primarily of a constriction rather than a dilation, as previously shown, with a smaller dilation near the switch moment itself. We conclude that the robust dilation previously found to follow perceptual switches was likely linked to the elevated cognitive effort brought about by the switches' task relevance, while the constriction that emerges when task relevance is removed is potentially due to visual cortical responses associated with perceptual switches. This evidence corroborates and extends previous work showing switch-related constriction in binocular rivalry and suggests commonalities between various forms of perceptual bistability with respect to neural mechanisms.

## Supplementary Material

Supplement 1

Supplement 2

## Footnotes

1 In the inversion-relevant condition, the median reaction time to triangle inversions was 0.72 seconds, while the median delay between OKN-based and manually reported switches that is evident from Figure 1C, right, was 1.11 seconds. The similarity between these numbers is consistent with the idea that OKN direction changes very shortly after a perceptual switch, whereas the key press follows after roughly a manual reaction time delay. The fact that the interval between OKN reversal and key response is somewhat longer, may be attributed to the difference in the task between the two conditions. Specifically, the transition between two percepts at the time of a switch is often gradual (see Naber et al., 2011) whereas the triangle inversions in our paradigm occurred instantly.

2 It should be mentioned that the original suggestion of norepinephrine involvement in perceptual switching (Einhäuser et al., 2008) was based, not just on the existence of switch-related dilations, but also on the finding that the magnitude of these dilations predicted the duration of the upcoming percept (switches with larger pupil responses were followed by longer percepts). Although this finding has been contested (Hupé et al., 2009), for the sake of completeness, we performed a similar analysis on our data. For each individual OKN-based switch, we quantified pupil dilation as the peak pupil size during a window from 100 ms to 3000 ms after the switch minus the average pupil size during the 100 ms before the switch. Correlating this value with the time delay until the next switch showed no significant relation in our data.
