# Supplementary material for: The pupil response to perceptual switches: What happens when you ignore them
Source: J Vis. 2025 Jul 3;25(8):5. doi: 10.1167/jov.25.8.5 (PMC12236628; doi:10.1167/jov.25.8.5)
Supplement: Supplement 1 [file jovi-25-8-5_s001.pdf]

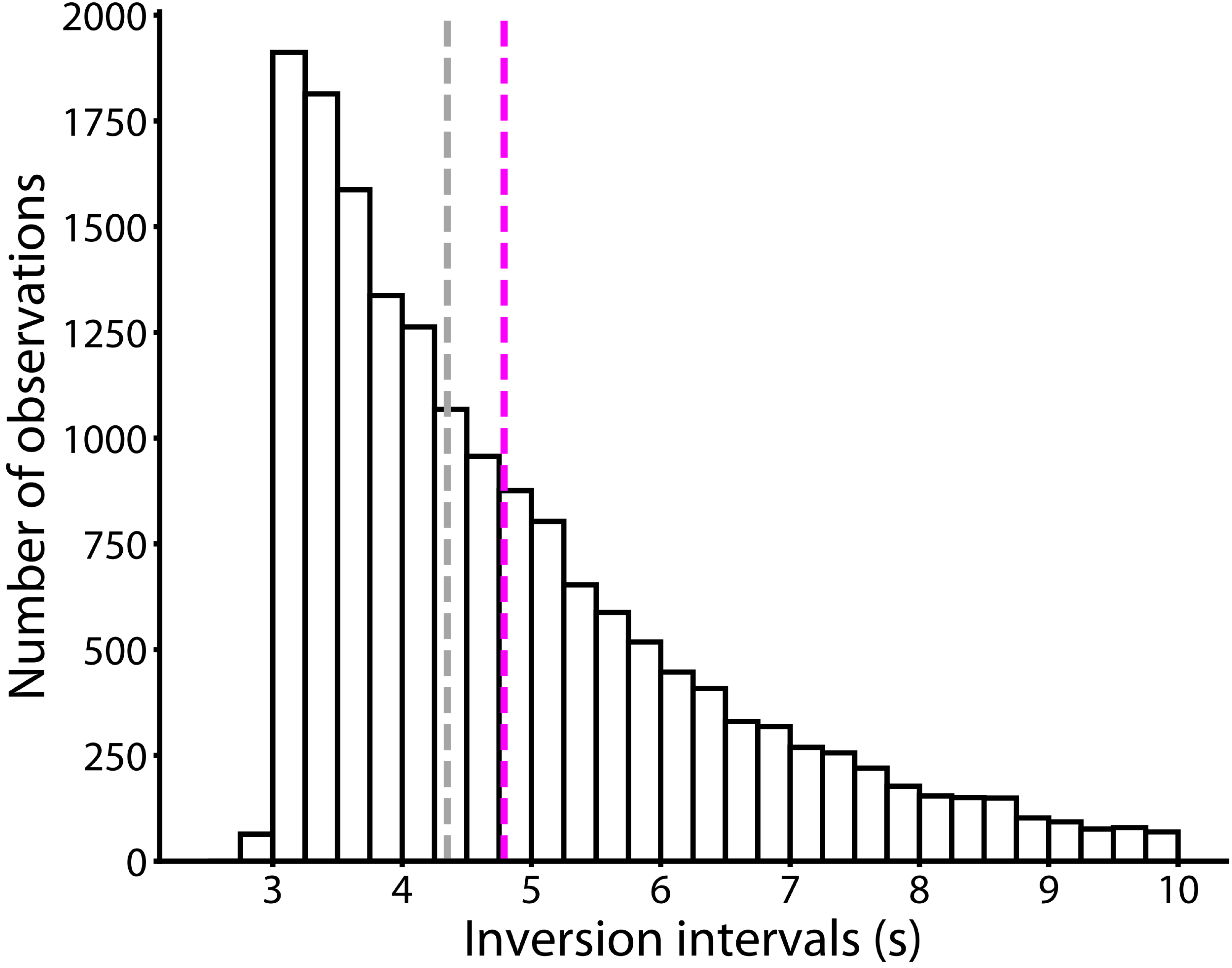

Supplementary Figure 1. The distribution of intervals in seconds between triangle inversions throughout the experiment. The dashed grey line represents the median (4.35 s), and the dashed pink line represents the average (4.79 s).
