# Supplementary material for: The pupil response to perceptual switches: What happens when you ignore them
Source: J Vis. 2025 Jul 3;25(8):5. doi: 10.1167/jov.25.8.5 (PMC12236628; doi:10.1167/jov.25.8.5)
Supplement: Supplement 2 [file jovi-25-8-5_s002.pdf]

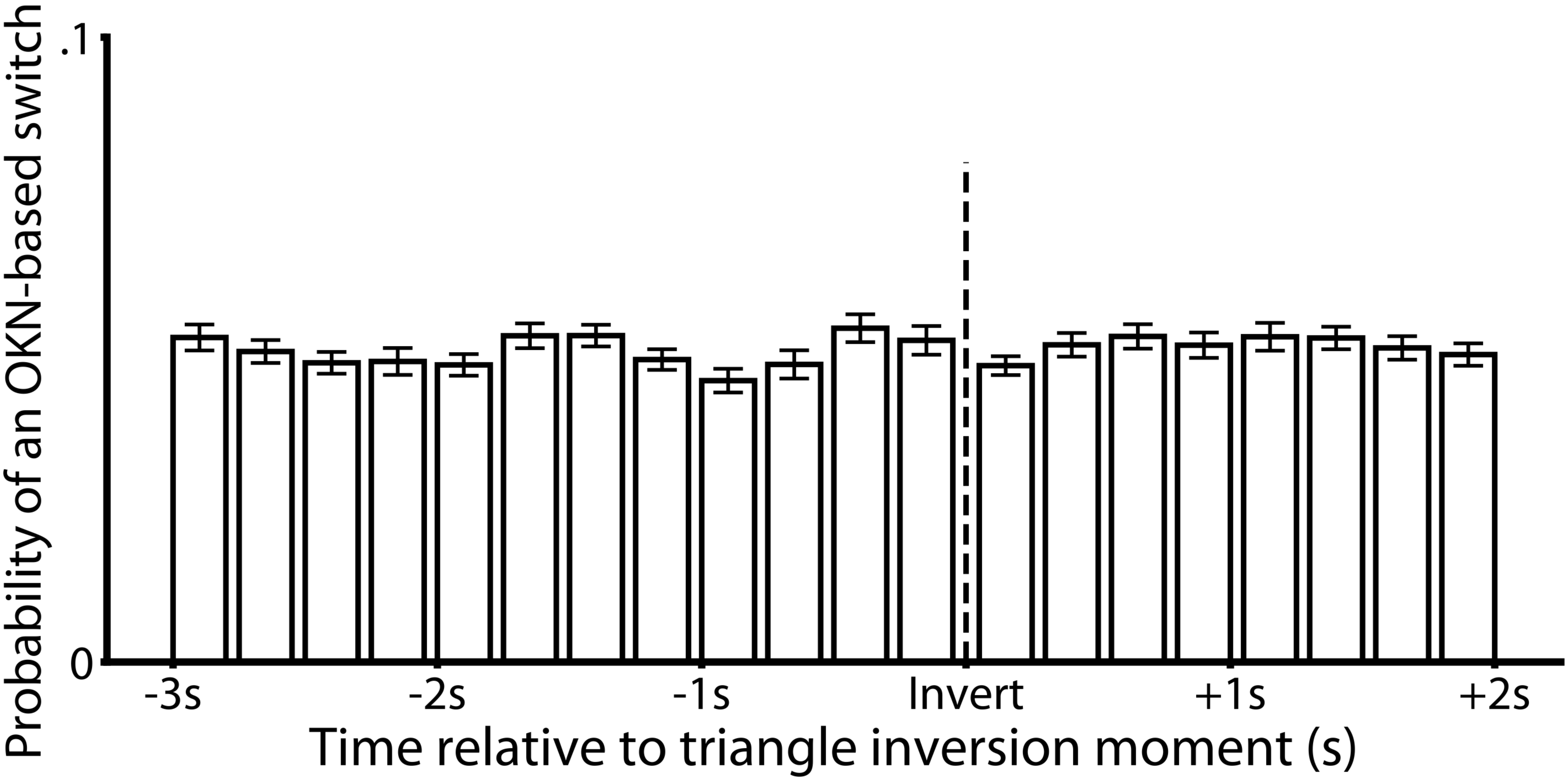

Supplementary Figure 2. The probability distribution of OKN-based perceptual switches relative to moments of triangle inversion. The error bars represent the standard error of the mean. Unlike Fig. 1C, the lack of a clear peak here suggests no distinct temporal alignment between OKN-based switches and triangle inversions.
